# Outlaw biker violence and retaliation

**DOI:** 10.1371/journal.pone.0216109

**Published:** 2019-05-08

**Authors:** Christian Klement

**Affiliations:** Department of Sociology and Social Work, Aalborg University, Aalborg, Denmark; Queensland University of Technology, AUSTRALIA

## Abstract

The number of outlaw bikers is growing globally. Despite this, little research exists on these groups and their alleged violent tendencies. To address this, the current paper uses unique data to examine whether gang violence causes outlaw biker violence. The period examined runs from mid-2008 until early 2012 during which violent clashes occurred between outlaw bikers and street gang members involved in an alleged conflict in Copenhagen, Denmark. A precise description of each individual act of violence would make it possible to identify whether specific acts were carried out in furtherance of the alleged conflict. This would allow one to determine whether outlaw bikers commit violence on behalf of their club. However, such knowledge is unavailable. The paper therefore takes a different approach by examining whether acts of violence committed by the two groups are statistically associated. In other words, it considers whether one or more acts can be described as retaliatory during the observation periods. The sample consists of 640 individuals involved with the Hells Angels Motorcycle Club or with non-biker street gangs–both of which are present in Copenhagen. Statistical models are used to predict 143 violent events committed by 196 outlaw bikers. The results suggest that violence committed by gang members predicts violence committed by outlaw bikers. This indicates that violent acts committed by outlaw bikers are at least partly a form of retaliation carried out on behalf of their club. The paper expands the literature on the kinds of inter-group, micro-level processes that can lead to reciprocal violence by including outlaw bikers in a literature that has previously focused on non-biker street gangs.

## Introduction

While the global number of outlaw bikers and biker clubs is unknown, their presence seems to be growing. One of the most prominent of these clubs, the Hells Angels Motorcycle Club, lists 466 chapters in 58 countries [1]. Another prominent club, the Bandidos Motorcycle Club, lists one or more chapters in 32 countries [2]. Both clubs originated in the US–the Hells Angels in 1948 and the Bandidos in the late 1960s, and the two clubs now have chapters in countries as diverse as Japan, Peru, Russia, South Africa, Thailand, Turkey, and Denmark, in addition to many other European nations. Despite their global presence, very little research exists on outlaw bikers and their alleged violent tendencies as noted elsewhere [3].

In a European context, outlaw motorcycle clubs are thought to be especially prominent in Germany and the Scandinavian countries [4]. Within Scandinavia, some consider Denmark the center of violent conflicts among outlaw bikers [5]. The first Danish chapters of the Hells Angels and Bandidos were established in 1980 and 1993, respectively [6].

From mid-2008 until early 2012, members of the Hells Angels and their associates allegedly clashed with four street gangs in and around the Danish capital, Copenhagen [7–8]. These violent clashes might have been partially sparked by a conflict over control of the Copenhagen cannabis market, though personal disputes may also have played an important role behind the scenes [7, 9–11]. The current paper examines whether Hells Angels members and their associates respond violently to violence committed by rival gang members. In other words, does violence committed by street gang members predict violence committed by members of the Hells Angels and their associates? If so, it would suggest that at least part of the violence committed by outlaw bikers was done as retaliation on behalf of their club. Given the paper’s focus on outlaw biker violence as retaliation, the nature of street gang violence is largely left unexplored since it is solely included in the paper in order to study outlaw biker violence.

The current study draws on two distinct types of data. The first is a list of 640 individuals whom police consider to have been either members of four Copenhagen street gangs or members/affiliates of the Hells Angels or Bandidos in or around Greater Copenhagen. The second set of data consists of criminal history and background information on the 640 subjects derived from the national statistical archive, Statistics Denmark. The combination of these datasets produces a wealth of information, which is rare in an international context when it comes to research on outlaw biker crime.

The statistical analyses indicate that street gang violence predicts outlaw biker violence. When gang violence reaches a specified level of intensity during a three-day period, individuals associated with the Hells Angels become more likely to engage in violence. This association remains despite controls on a host of rival causes and achieves more statistical significance during a period of heightened inter-group conflict than outside this period. It seems that Hells Angels members and their associates respond violently as a group and that their violence is not only an individually directed activity.

The paper draws on theoretical frameworks developed in the expansive literature on gangs and violence as will be explained in the section below. It does so broadly by placing the paper within the literature on micro-level processes that facilitate gang offending. It also does so more narrowly by using a theoretical model developed to describe cycles of gang violence in order to explain the patterns identified in the analysis. The analysis tests whether violence committed by one group increases the risk of violence being committed by another. In this way, it tests whether a crucial part of the mentioned model of gang violence is supported by the data on the conflicts between outlaw bikers and gang members. With this theoretical setup and based on its analysis, the paper expands the literature on inter-group micro-level processes that might lead to reciprocal violence. Until now, the majority of this literature has been largely based on studies of street gangs. The current study adds an example of reciprocal violence committed by outlaw bikers to this research tradition.

Outlaw motorcycle clubs are sometimes referred to as *outlaw motorcycle gangs*, abbreviated *OMCG’s* (for instance in [3, 12]). Depending on the definition of an outlaw motorcycle gang and the particular club in question, this may or may not be an appropriate term. In the current study, the term outlaw motorcycle club is predominantly preferred. The current study also contributes to the debate on how to categorize outlaw motorcycle clubs. It can, after all, be argued that if members of an outlaw motorcycle club and their associates commit violence on behalf of the club, the term *outlaw motorcycle gang* may indeed be more appropriate.

## Theory

It is well-established that gang members engage in high rates of offending and that this pattern is attributable to both selection and facilitation [13–15]. In short, not only are gang members intrinsically more likely to offend even before they become affiliated with a gang (selection), their likelihood of offending is also increased by this affiliation (facilitation). The facilitation effect is found in a majority of methodological contexts, as pointed out by Pyrooz and colleagues in their meta-analysis of 179 studies of the relationship between gang membership and offending [16]. Yet, as these authors also point out that “*…the precise mechanisms of this relationship still remain unknown*.” (page 384 in [16]). Based on their meta-analysis of studies from 17 countries, Pyrooz and colleagues conclude that the relationship between gang membership and offending in general is weaker in a European and Latin American context than it is in the US [16]. Whether the stronger relationship identified in the US can be attributed to different levels of gang organization, different socio-cultural conditions, or a combination of the two also remains unknown. The lack of knowledge on the facilitation processes in general as well as the weaker relationship between gang membership and offending in Europe as compared to the US increases the relevance of the current study which examines the facilitation of gang violence within a European context, i.e., whether reciprocal violence can account for increased levels of violence.

Pyrooz and colleagues add an additional observation that is relevant to the current study. Even if numerous individual factors are taken into consideration, the relationship between gang membership and offending is only partially accounted for in their meta-analysis. This suggests that to fully explain the relationship statistically, non-individual factors need to be taken into consideration [16]. This corresponds well with the notion that gangs are groups. To fully account for their collective behavior, including their violent behavior, micro-level processes within and between gangs need to be taken into account as this is necessary in order to explain the facilitation relationship between gang membership and offending [17–19]. This observation further increases the relevance of the current study which examines the reaction of outlaw bikers to gang violence.

Although the focus of the current study is on micro-level processes *between gangs*, a few words should be said on *within-gang* processes because the two are likely to intercept. Micro-level processes related to facilitation are perhaps best represented by McGloin and Collins who identify four general types of intra-gang micro-level processes [20]. The first of these four types deals with changes in criminal opportunities following gang induction. Joining a gang typically implies a shift in lifestyle circumstances, which may increase opportunities for crime [21–22]. This idea is based on a routine activities perspective and argues that when adolescents socialize in unstructured and unsupervised settings, natural inducements for delinquency can present themselves and tempt even pro-social youth to engage in deviant activities [23–24].

The second of these four types of intra-gang micro-level processes concerns the effect that the presence of others can have on individual decision-making[20]. In its essence, the argument is that individuals act differently in the presence of others than they would on their own. This has to do with de-individuation processes such as the diffusion of responsibility. Others become part of the individual decision-making process which facilitates collective behavior, including delinquency, and implies an interdependence in individual decision-making [25]. This corresponds with the empirical finding that adolescents are more likely to engage in risky behavior when peers are present [26]. It also corresponds well with the finding that at least officially recorded violent delinquency tends to include a larger proportion of unique offenders than the numbers recorded for non-violent offenses [27].

The third type of intra-gang micro-level process that may facilitate criminal involvement has to do with social status [20]. The general idea is that the gang serves as a context or medium in which social status is achievable through delinquency. For example, demonstrations of toughness are highly rewarded when gang peers serve as an audience [28]. The relationship between delinquency and social status within a gang setting is amplified in comparison to what it would be in other youth group settings. This is because the gang offers marginalized youth an opportunity to achieve a social standing that is otherwise not easily achievable, as has been persistently pointed out by gang researchers [29–33].

The fourth and final type of intra-gang micro-level process emphasizes that normative influence in gangs usually supports delinquency, including violence [20]. In gangs, behavior might be imitated, reinforced or sanctioned in order to be in accordance with the norms of the group or provide models for identification [34–36]. More recently, gang membership has been conceptualized as a turning point in new members’ lives where individual behavior is seen to be affected by such processes [15, 37–39]. The normative influence is channeled through the identification with gangs: the stronger the identification with the gang compared to the identification with other groups, the stronger the normative influence. If in-group/out-group distinctions are prominent and the investment in, and commitment to, group identity is high, then conformity with group norms will also be high [40]. However, the relation between cohesion and delinquency can also run the other way. Competition for status within a delinquent subculture can cause a status-threatened gang leader to resort to violence in order to improve his standing within the group and increase group cohesion [18, 41]. This points to the fact that a gang usually exists in relation to other gangs.

The micro-level processes at work between gangs might be an important part of the full explanation as to why gang membership may facilitate offending, as noted by McGloin and Collins [20]. This may be particularly applicable to cases of violence between gangs–which are often of a reciprocal nature. This is perhaps best conceptualized by the cycle of gang violence model proposed by Decker [42] and expanded by Decker, Melde, and Pyrooz [17]. The model captures a typical violent micro-level process between gangs and is based on Decker’s three-year fieldwork study of gang members and their violence in St. Louis, USA. The cycle involves at least two gangs and a series of expected steps, the last of which (Step 5) generally sets the stage for actors to pursue one of three directions. The five steps are as follows:

1. In the initial stage of the cycle, gang members have loose ties to their gang. In other words, they are not closely integrated in the gang;
2. A real or imagined external threat from a rival gang is collectively identified. This could happen; for instance, through rumors and/or a symbolic show of force. The identified threat increases the importance of violence, expands the number of participants, and reinforces group cohesion;
3. An event mobilizes gang members. This does not have to include violence, but could involve violence;
4. Gang activity escalates;
5. A violent event occurs. The violence is either directed at the rival gang or can be interpreted to be so.

After step five in the cycle, three developments are possible [17, 42]:

- The violence leads directly to de-escalation;
- An outside intervention occurs and leads to a de-escalation;
- The rival gang retaliates and allows the cycle of violence to continue (repetition of steps 4–5).

Empirically, violence has been identified as having this reciprocal or contagious potential, not only in Decker’s original fieldwork study, but also in other studies [43–49]. A desire for revenge and an unwillingness to accept loss of face can all motivate a group to retaliate, especially if an audience is present. Retaliation may not only be expressive, but also instrumental. A wish to deter further aggression and maintain a market foothold in, for instance, the drug business might also motivate retaliation [48, 50–52]. However, as has been pointed out, empirical evidence suggests that the distinction between expressive and instrumental motivation tends to be blurred [53]. The most illustrative case is perhaps that of gang territory. Territory is not only important because it constitutes identity and an economic base; it is also an essential determinant of social status and dominance. This is why disputes over territory are not only concerned with maintaining a market foothold, but also with dominance or, as Papachristos writes: “*…turf’s symbolic value is contingent on the ability of a group to fight and avoid subservience to other groups*.” (page 117 in [53]). In the current study, this is also the case, which implies that expressive and instrumental violence will not be identified separately. The overlap between expressive and instrumental violence might apply in particular to outlaw motorcycle clubs because they are territorial in essential aspects. If a competing gang is successful in establishing itself in a given territory, presumably for the purpose of getting a share of the local illegal market already claimed by another club, the previously established club risks losing face, city dominance, and its local market share.

The distinction between expressive and instrumental offending has also been noted in the sparse literature on outlaw motorcycle clubs [54]. Based on a general reading of this literature, four distinct types of crime have been elaborated in an attempt to categorize crime committed by outlaw bikers [55]. The first type is comprised of spontaneous expressive acts conditioned by situational factors such as those present in a typical bar fight. The second is made up of planned expressive acts that are usually violent and directed at rival groups. In such cases, expressive and instrumental motivations overlap. The third type of crime concerns short-term instrumental acts. They often involve only one or a small number of club members who take advantage of unique opportunities or act in response to the needs of one or more group members. This third category of crime includes acts may vary on a continuum from planned to spontaneous. The fourth and final type of crime includes ongoing instrumental enterprises. These acts involve one or more cliques of members, are designed to result in a profit, and are often carefully planned in advance. Such criminal acts could be the production or distribution of drugs. The insight relevant to this study is that both the literature on gangs and the literature on outlaw bikers point out that even though it might be difficult to separate the two aspects empirically, violence between groups has the potential to be expressive as well as instrumental.

Although it does not draw on the selection and facilitation framework from the gang literature [13–15], part of the limited literature on outlaw motorcycle club membership and criminal behavior does consider the relationship between the two. Yet, only a few studies have systematically considered this connection [54, 56–61]. Only one of these studies has longitudinal observations of gang members before and after their gang affiliations are established as well as a comparison group–the combination of which makes it possible to examine the causal direction between outlaw motorcycle club affiliation and heightened criminal involvement [59]. While Alain finds that incarcerated outlaw bikers do not differ significantly in terms of crime frequency, violence, or seriousness of offending when compared to the general population of inmates in Quebec and Canada [56], most other studies indicate the presence of an association between outlaw motorcycle club affiliation and heightened criminal involvement, including violence. It should be noted, however, that none of these studies are able to capture between-group micro-level processes such as those hypothesized to operate within in the cycle of gang violence model [17, 42] and found in gang studies [43, 46–47, 49]. The absence of studies of micro-level processes between groups in prior research increases the relevance of the current paper.

The criminological literature on retaliation between groups, regardless of whether it is expressive or instrumental, has primarily been based on studies of groups characterized as street gangs [43, 46–47, 49]. In this paper, the literature is expanded to include acts of possible retaliation committed by individuals affiliated with outlaw motorcycle clubs. The current study does not attempt distinguish between expressive or instrumental motivations for retaliation. Furthermore, the current study does not make any presumptions as to whether individual acts of violence have been carried out on behalf of the offenders themselves or for the sake of their club. Given the available data, this is unavoidable since such a categorization would require unavailable insight into the circumstances of each individual act of violence. Instead, the question as to whether acts of violence committed by outlaw bikers is retaliatory and committed on behalf of their club is explored by interpreting the level of statistical association between acts of violence committed by members of the groups. This does not test any of the four general types of intra-gang micro-level processes highlighted by McGloin and Collins [20] and explained above. It does, however, indicate to what degree violence committed by gang members increases the risk of violence committed by outlaw bikers. This can be seen as a test of whether a crucial part of the cycle of gang violence model outlined by Decker and colleagues [17, 42] is present in a context in which the involved groups are not all street gangs.

The study presupposes the presence of groups within a limited period of time and within a limited geographical area. In the following, it will be rendered plausible that these conditions are present in the current study.

## An introduction to outlaw motorcycle clubs and the related terminology

Outlaw motorcycle clubs are defined as motorcycle clubs that have not been registered with a nationwide motorcycle association, i.e., the American Motorcycle Association [62] or its European counterpart the Federation of European Motorcyclists’ Associations. The Hells Angels and Bandidos Motorcycle Clubs both count as such and have been present in Denmark since 1980 and 1993, respectively. The current study includes individuals active within the Hells Angels and the Bandidos, as well as their support and puppet clubs. Support and puppet clubs are included since they play an important part in the overall pattern of violent crime attributable to Danish outlaw bikers [8]. Individuals affiliated with the Hells Angels, the Bandidos, or any of their support or puppet clubs are referred to as *HAMC individuals* and *BMC individuals*, respectively.

Their allegiance to competition, subcultural values, and structural organization may help explain why outlaw bikers are likely to engage in violent conflicts. An important feature of the subculture is formal and informal competition between individual outlaw bikers and between their clubs. It provides an opportunity to demonstrate subcultural values. Such competition is well-established and provides a path to high social standing [63–64]. It can, however, also trigger violent conflicts between clubs [63–64]. In addition, besides placing a high value on motorcycling, outlaw bikers put a high value on fellow outlaw bikers, especially those within one’s own club, and on traits associated with traditional masculinity, e.g., being honorable, tough, strong (though not necessarily in physical terms), independent and non-submissive [62–64]. Most outlaw motorcycle clubs–if not all–deny females membership. While women certainly appear in the subculture, they almost always do so in submissive roles [62]. Also, emotional bonds to women could detract from club obligations and loyalty. Inclusion of women in membership roles would also threaten the subculture’s exclusively masculine identity.

Outlaw motorcycle clubs might be more able to act collectively than more informal groups because they are organized bureaucratically [3, 55, 62–63, 65]. Within chapters there are specialized roles for members and a hierarchy ranging from hang-arounds and prospects at the two lowest levels to a president at the top [62]. Positions immediately subordinate to the president generally include a vice-president, a sergeant-at-arms, a secretary/treasurer and a road captain. The clubs usually have written rules including a club constitution [55, 62–63]. Admission to club membership is generally a multi-step process involving background checks, member sponsorship, a probationary period and final approval by membership vote. As in the police and military, a certain degree of uniformity in appearance is mandatory (dress, colors, and motorcycles) and club chapters have clearly defined geographical areas of authority [62]. The name of the territory appears on a chapter’s patch. A patch is worn on the back of a vest which for full club members usually consists of club name at the top, club symbol in the center, and the name of the chapter’s territory at the bottom [62]. Unlike most bureaucratic organizations, some outlaw motorcycle clubs admit new members—and elect existing members to formal positions–only on the basis of majority or unanimous vote [62].

Together the subcultural values, including the willingness to compete, and the formal organization produce and reproduce collective identities within a closed, highly cohesive, hierarchal society [3, 62–63]. Taken in combination, these attributes make the willingness to engage in violent conflicts more likely and reinforce the capacity to do so, and fit well with the expectation that outlaw bikers engage in violent retaliation.

## Causes and context of the conflict in Copenhagen

Outlaw bikers and their predecessors have been active during several periods in Copenhagen’s recent history. In the 1980s a conflict between the Bullshit Motorcycle Club (founded 1979) and the Hells Angels (founded 1980) resulted in 13 deaths and the establishment of the Hells Angels as the dominant club [6]. Three years after two local clubs were given permission to form the first Danish chapters of the Bandidos in 1993, the so-called Great Nordic Biker War spread to Denmark with Hells Angels and Bandidos on opposing sides. After five individuals were killed in Denmark, the entire Scandinavian conflict was resolved in 1997 by agreement between the two clubs [6]. The content of the 1997 agreement was never made public. Nonetheless, it is alleged to have divided all major Danish cities into three groups: Hells Angels cities, Bandidos cities, and so-called open cities. Copenhagen was supposedly one of the Hells Angels cities.

The period 2008 to 2012 was characterized by a series of violent events involving the Hells Angels and four Copenhagen street gangs: the Blagards Plads Gang; the Tingbjerg Gang; the Mjolnerparken Gang; and the Brothas Gang [7]. The focus of the current paper is whether some of the outlaw biker violence committed during this period can be classified as retaliation.

**Fig 1:**
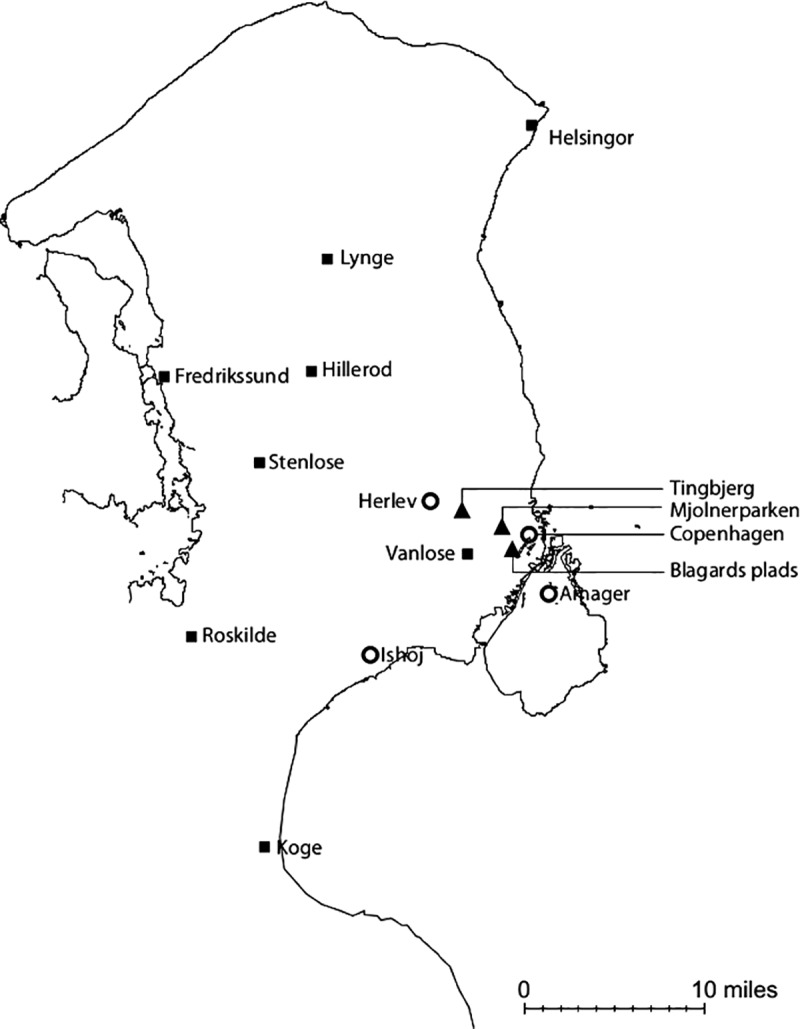
Map with locations associated with HAMC individuals, the Copenhagen street gangs, and BMC individuals in or near greater Copenhagen^A^. Map note A: Hells Angels, Copenhagen street gangs, and Bandidos locations are represented by circles, triangles, and squares, respectively. For the outlaw motorcycle clubs, each location can represent more than one chapter. Seven locations associated with 20 individuals have been removed from the map to protect anonymity.

These four street gangs are based in three Greater Copenhagen areas: Blagards Plads, Tingbjerg, and Mjolnerparken (see Fig 1). The Brothas Gang–whose name is not geographically derived, but rather comes from the English word *brothers*–has its roots in Tingbjerg and Mjolnerparken [7].

Two events especially set the stage of the conflict. First, in 2007 the Hells Angels created a puppet club they named AK81 [7]. “AK” is an abbreviation for the Danish words “Altid klar” (“Always ready”), while 8 and 1 correspond to the letters HA (i.e., Hells Angels) in the Roman alphabet. AK81 members are not required to own motorcycles and are considerably younger than most Hells Angels members [8]. The second important event occurred on June 30, 2008, when a spokesperson for the Hells Angels published a text titled the *Jackal Manifest*. According to it, youth who commit crime, act in a cowardly fashion, suppress women, terrorize others, despise Denmark, and generally behave like jackals [66]. The text claims that such youth are predominately found among individuals with Arabic and Islamic backgrounds. It closes by concluding that one never ought to give in to such “*…human waste*!” (Author’s translation from [66]). At this point it is important to note that 97% of people officially recorded as outlaw bikers in Denmark are ethnic Danes, whereas gang members are mostly either immigrants (44%) or second-generation immigrants (39%), and the remainder are ethnic Danes (17%) [8]. While the general picture may have changed since then, the figures cited are based on 2009 data from the Danish National Police and Statistics Denmark and are therefore directly relevant to the 2008–2012 conflict at issue in this paper. The publication of the *Jackal Manifest* in 2007 is important because it may have attracted individuals with anti-immigrant sentiments to the Hells Angels and thereby served to increase racial tensions between Hells Angels individuals and members of the four street gangs. It is worth noting that in the United States, clubs such as the Hells Angels and Bandidos are also predominantly comprised of white males [65].

Two incidents sparked the beginning of the 2008–2012 conflict between the Hells Angels and the four street gangs. On 16 July 2008, several shots were fired in the Copenhagen suburb of Vanlose [7]. A month later, on 14 August 2008, a 19-year-old second-generation immigrant youth was shot and killed in Tingbjerg, another Copenhagen suburb. It turned out to have been a friend of his standing nearby that was the intended target. Later on, three AK81 members were charged and convicted for these two shootings [7]. These two incidents mark the beginning of the conflict between the Hells Angels and the four street gangs.

Police and the media have at least three sensible hypotheses concerning the cause of the conflict. The first hypothesis focuses on personal conflicts. During the trial of the AK81 member convicted of murdering the 19-year-old youth in August 2008, it was revealed that the intended victim and the AK81 member had engaged in previous fights and even gun battles because of personal disputes [7].

A second hypothesis as to the cause of the conflict focuses on efforts to control the illegal drug market. Members of the groups seem involved in these markets as suggested by their frequent convictions for drug-related offences [8]. The series of police crackdowns on the open-air cannabis market in the Copenhagen neighborhood of Christiania in 2004 created instabilities that might have resulted in some street gangs increasing their share of the Copenhagen cannabis market. The police operations prompted buyers to seek out new sellers in other parts of Copenhagen [9]. Bigger market shares and the associated profit might have made the gangs stronger competitors to the Hells Angels who had controlled the Christiania cannabis market since at least 1997 [9]. The National Police has explicitly noted that the Blagards Plads gang has taken over parts of the illegal drug market previously controlled by the Hells Angels [10].

A third possible cause of the conflict between the Hells Angels and the opposing street gangs relates to competition for subcultural status [7, 11]. However, as was noted in the theoretical section above, distinguishing between factors related to social standing and factors related to economic interests is difficult.

While the exact strength of each cause is unknown, all three potential causes may have contributed to the conflict in varying degrees. As described in the above, personal rivalries may have played an important role during the initial phases, whereas the impact of competition over illegal market share, dominance, and status may have increased as the conflict progressed.

It is unclear exactly when the conflict ended. However, on 21 April 2012, Hells Angels and AK81 members walked through one of the main shopping streets of Copenhagen with their previous adversaries, the Brothas Gang. It is estimated around 120 individuals participated in this walk which was interpreted by the police and the media as a show of combined force [67]. A few gang-related confrontations were registered during the following weeks, though none of them seem to have involved the Hells Angels.

## Two data sources

The empirical part of this study is based on data from the Danish National Police and Statistics Denmark. The National Police provided information in June 2009, October 2012, and October 2013. This information concerned 2,647 unique individuals suspected of associating with groups involved in serious violent crime and/or the distribution of illegal drugs. The 2,647 individuals include persons affiliated with the Hells Angels, Bandidos, their respective support and puppet clubs, as well as members of the four Copenhagen street gangs that opposed the Hells Angels in the 2008–2012 conflict. The data come from the Police Intelligence Database (PID) where individuals related to outlaw biker and street gang crime are registered. PID data include national identification number, club/gang affiliation and *PID date* (i.e., the date an individual was first recorded in PID). Police squads specialized in outlaw bikers and street gangs and regular police patrols gather information and report it to the National Intelligence Center. Each piece of information is assessed in terms of investigative value and recorded in PID depending on the results of that evaluation. According to the National Police, affiliation with an outlaw motorcycle club is sufficient grounds for registration in PID [8, 10]. This practice is based on the assumption that in order to be affiliated with an outlaw club one essentially has to be involved in serious crime. The decision as to whether an individual suspected of street gang affiliation should be registered in PID is more difficult. This is because PID registration in this case requires that the individual be personally suspected of involvement in serious crime. Furthermore, gang affiliation must be substantiated. It is important to note that persons can be registered in PID without having been charged for or convicted of a crime.

The National Police claim to remove people from the database after two years if they are not suspected of any new crimes [60]. This is, however, only relevant for gang members because the National Police consider an outlaw motorcycle club affiliation as sufficient reason for PID registration. Thus a registered outlaw biker will only be removed from PID if he ceases his outlaw biker affiliation.

The national identification numbers available in PID make it possible to link additional data on all individuals from the national statistical archive, Statistics Denmark. Statistics Denmark is able to provide extensive information on anyone who has had their official residence in Denmark at any time since 1980. Although some of them overlap, Statistics Denmark currently lists 323 data registries on their web page [68]. From these registries, it is possible for researchers in Denmark and their collaborators elsewhere to obtain data for research purposes provided they pay the costs and obtain the proper permissions from Statistics Denmark. The registries are based on administrative data and cover subjects such as health, labor market, family type, education, trade, and crime. The data in the crime registries in Norway, Finland, Sweden, and Denmark are considered of high quality as noted by Lyngstad and Skardhamar [69].

The current study combines PID data from the Danish National Police with data from six of the 323 registries maintained by Statistics Denmark. Crime data was obtained from the following three registries (Danish: *Kriminalstatistik afgørelser*, *Kriminalstatistik konfererede sager*, and *Kriminalstatistik sigtelser*). Data on educational attainment, employment status, and family type was obtained from three additional registries (Danish: *Registerbaserede arbejdsstyrkestatistik*, *Familieforhold*, and *Højeste fuldførte uddannelse*). The PID data, including the national identification numbers, was handed over to Statistics Denmark which replaced each national identification number with a unique, anonymized replacement number. The replacement number was generated using a numerical key that only Statistics Denmark possesses. Statistics Denmark used the same numerical key to replace the national identification number of the individuals in the data obtained from the six registries. This means that each individual has a unique identification number in all the resulting datasets by which information can be linked. The resulting datasets were made available for analysis on Statistics Denmark’s secure server.

This study assumes that the PID sample is roughly representative of persons involved on the Copenhagen outlaw biker and street gang scenes during the period of observation. If this assumption does not hold, other assumptions about the causal mechanisms at play break down. However, outlaw biker and street gang crime has a high priority for law enforcement in Denmark. A ranked set of priorities for the Danish Police and the Public Prosecution Service is outlined in a *performance contract*. The performance contract identifies a handful of priorities and assigns a weight to each. The sum of these weights adds to 100%. The performance of both the Danish Police and the Public Prosecution Service is measured according to these general priorities. Outlaw biker and street gang crime was given a 20% priority rating in the performance contract of the National Police for 2012 and can thus be seen as a top priority for the Danish Police [70].

The tables, figures and models below are based on a subset of the data that includes all registered members of the four Copenhagen street gangs and all members and affiliates of the Hells Angels and Bandidos motorcycle clubs *in or around the Greater Copenhagen Metropolitan Area* (hereafter Greater Copenhagen). The data are limited to violent events in which the individuals involved were ultimately convicted.

## The sample and its characteristics

This section explains the reasoning and means by which the time periods and sample analyzed in the current study were chosen. It also reflects on the validity and reliability of the data, ethical considerations in connection to the setup of the study, and provides descriptive statistics on the sample. This is done in order to make the basis of the study as transparent as possible.

### Delimitation of the periods of analysis and the sample

This analysis defines the *Conflict Period* as 6 July 2008 to 21 April 2012. The designated start date is ten days prior to the first reported, conflict-related shooting on 16 July 2008. The ten-day run-up is included because some of the regression models portrayed below require taking violent events within the previous ten days into account. 21 April 2012 is designated as the end date because this is when the show of combined force by HAMC individuals and their former adversaries from the Brothas street gang took place. Using this end date also protects against the inclusion of other conflicts that erupted after this date which might otherwise bias the results. A second, somewhat extended period is also examined below. This period runs from 10 June 2007 until 30 June 2013. Its start date is set precisely two years prior to the date when the last individual was registered in the first PID data set received for analysis (in June 2009). This is important because of the National Police’s aforementioned (stated) policy of deleting people from the database if two years pass without suspicion of new crimes. The end date, 30 June 2013, is chosen because there is a lag between the date a crime is committed and the date it is dealt with by the legal system. In the current sample of registered violent events, 56% resulted in a court decision within six months of the act. The Extended Period represents a time frame within which information on persons and violent events are sufficiently detailed for reliable analysis.

**Fig 2:**
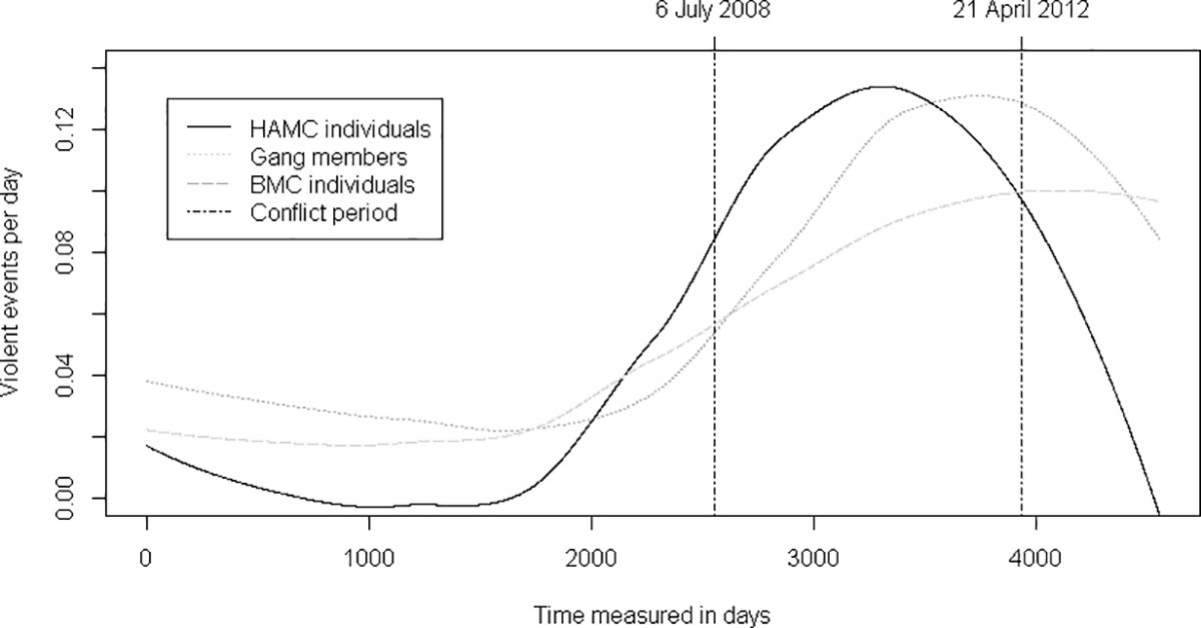
Convictions for violent crimes per day across groups over time (n = 793).

The sample consists of 196 and 228 individuals in the Conflict and Extended periods, respectively. In both periods, approximately half the sample is affiliated with the Hells Angels (55% and 48%) while approximately a quarter is connected to Hells Angels support clubs (20% and 25%) and another quarter to Hells Angels puppet clubs (26% and 26%). In Fig 2 smoothed curves represent violent events for the three groups during the period 10 July 2001 to 31 December 2013 (applying the R curve smoothing function LOESS). The figure includes only violent events involving persons who were registered in PID at the time of the act. The two vertical lines demarcate the 2008–2012 Conflict Period.

A considerable share of the violent events portrayed in Fig 2 took place within the Conflict Period. Violent activity for both HAMC individuals and street gang members peaked within that period. A significant proportion of the violent events involving BMC individuals also occurred within the time frame of the Conflict Period–despite the fact that the Bandidos were not specifically involved in the conflict. Periodic removal of (seemingly) criminally-inactive individuals from PID may also influence the shape of the figure since violent events are only included when committed by persons registered in the PID database.

### Reflections on the validity of the sample

The unobserved *dark figure of crime* haunts not only the study of ordinary street crime, but also the study of gang violence. In the United States, police data on gang homicides have been validated using multiple measures and have been shown to be sufficiently valid for research, especially in cities with specialized police gang squads, as pointed out by Decker and Pyrooz [71]. In a continuation of that multiple measures research, Katz and colleges found that police data on gang homicides in the United States exhibit a high degree of reliability for jurisdictions with populations over 200,000 inhabitants [72]. Furthermore, research finds no evidence that US law enforcement officers systematically inflate estimated levels of gang homicide [73]. Similar research is not yet available in a European context. The most pertinent European study might be a recent comparison by Rostami and Mondani of the use of three types of data to measure local street gang activities in Denmark’s neighboring country of Sweden. The data sources include intelligence-based police data, general police surveillance data, and co-offending data [74]. The co-offending data are a combination of police intelligence data and data from governmental records similar to the PID data used in the current study. While the Swedish study focuses on social network analysis, there are important parallels to the current study. Though not without their own weaknesses, the co-offending data are probably the most valid of the three data sources [74]. It is also worth noticing, that the Danish National Police seems to make sure that the information in PID is kept up to date. Out of the total number of outlaw bikers recorded n PID in June 2009, October 2012 or both, only 33% were recorded in both June 2009 and October 2012, while 22% and 46% were recorded either June 2009 or October 2012, respectively [75]. Based on that, it seems that individuals are routinely added as well as removed from PID database. Given the two scholars’ conclusion based on Swedish data similar to the data in the current study, the presence of specialized police squads in Greater Copenhagen, and the capital’s population of over 1 million inhabitants, and the routinely recording and removal of outlaw bikers in PID the PID data are assessed to be sufficiently valid and reliable for analysis. Note also, that the information in the analyzed data have been validated to such a degree that violent offenders have been convicted in a court case.

### Ethical considerations

The individuals analyzed in this study have not been asked, nor given their consent, for participation in the current research. Furthermore, the results of this study might possibly offend at least some sample members. One could even argue that the study’s results might change public opinion for the worse, and thereby harm sample members. All of this said, there are important reasons for having conducted the study in the way it was done. First, it would probably have been difficult, if not impossible, to have carried out the study using any data other than administrative crime data employed herein. There is at least one study of outlaw bikers that uses a self-report survey (implying consent), but the validity of the survey responses seems highly questionable [58]. That study relied on an incarcerated sample and drew its sample of “outlaw bikers” on the simple basis of an expressed preference for the use of motorcycles over cars. Given the biases likely to arise from self-report surveys in this context, the current study saw no alternative but to use administrative data. Second, the use of official registry data for research is strictly regulated by Statistics Denmark. Researchers need to go through a stringent application process and receive approval from Statistics Denmark in order to gain access to the official registries. The *Law on Statistics Denmark* [76] provides the legal framework surrounding the use of official registry data for research purposes. Results based on official registry data provided by Statistics Denmark are generally only allowed to be published if they are aggregated to such a level that individual subjects can never be identified. However, given the particularly sensitive nature of the current data, Statistics Denmark only allowed its use pursuant to stricter guidelines regarding the publication of results. These guidelines required that the results should not only be aggregated at an individual level, but also at a group level. This explains why all published results in the current study are based on individuals from two or more groups combined. For example, the results under the headings “HAMC individuals” and “BMC individuals” pertain not only to individual members of the two clubs, but also individual affiliates from their support and puppet clubs. Likewise, the results under the heading “Gang individuals” are based on data from the combined membership of all four Copenhagen street gangs. Statistics Denmark has not placed any additional restrictions on the publication of the results. Furthermore, as required by Danish law, the current study has been reported to the Danish Data Protection Agency. The faculty, where the study was originally conducted, has also been made aware of the study’s use of confidential data. The Danish National Police was legally able to provide the PID data for research purposes based on the statutory order regulating PID [77]. Third, the vast majority of studies based on official crime data fail to ask sample members for their consent even though the results of these studies could, in theory, change public attitudes toward some of those sample members in a negative way. While the fact that “everyone does it” is not an argument for continuing that same practice, it does imply that the current study is no different from the vast majority of similar studies in this regard. Fourth, while negative attitudes towards outlaw bikers might increase as a result of the current study, this must be balanced against the knowledge that the current study provides that might help to prevent future violence and thereby prevent harm.

### Descriptive statistics

Table 1 shows the number of convictions for violent crimes committed by members of the three groups during the Conflict and Extended Periods, respectively. *Number of convictions* pertains to events as opposed to individuals. Multiple individuals can be charged in connection with a single event. *Number of dates* is the number of days on which violent events occurred. The number of violent individuals, court cases, and dates are shown in Table 1 to give an impression of the context in which the violent crimes take place and their independence as observations.

**Table 1:** Convictions for violent crimes, by group and period.

Type of violent event / Sample n	HAMC individuals		Gang individuals		BMC individuals	
	Conflict Period	Extended Period	Conflict Period	Extended Period	Conflict Period	Extended Period
Arson	7%	6%	15%	12%	4%	3%
Crimes against personal liberty	7%	7%	7%	6%	7%	5%
Homicides and attempted homicides	18%	15%	15%	14%	9%	6%
Robberies	6%	7%	13%	13%	10%	11%
Threats	10%	12%	11%	13%	25%	25%
Assault	53%	53%	39%	42%	46%	49%
Number of PID-active individuals	196	228	160	263	284	358
Number of violent individuals	69	86	52	63	76	100
Number of court cases	82	113	67	91	87	127
Number of dates	88	121	77	103	96	146
Number of victims in opposing group(s)^A^	< 10	< 10	< 5	< 5	< 5	< 5
Number of convictions	171	220	109	148	163	231

A: The data on Hells Angels victimizations include both Bandidos victims and Copenhagen street gang victims. Meanwhile, the victimizations committed by the Bandidos and the Copenhagen street gangs are only against HAMC individuals and in no case (in these data) against each other.

The dependent variables in the models below consist of 143 and 187 violent events. In Table 1 these events are represented by 171 and 220 violent events. Multiple violent acts carried out by the same individual on the same date are coded as a single violent event because the dependent variable in survival models is binary.

There are two explanations for the relatively low number of victims in opposing groups. First is the reluctance of people in this environment to report victimization. After all, openly involving the police risks exposing one’s own illegal activities, being labeled as a snitch, and/or showing signs of weakness: “*…asking for help makes it apparent that one cannot handle his or her own business*.” (page 42 in [50]). Consequently, a proportion of violent events probably never find their way into official police statistics.

A second explanation for the seemingly low number of victims relates to the nature of the available data. People who die are systematically removed from PID. Such people will no longer show up in the database and therefore no longer count as victims. While this explanation is primarily relevant to homicide, it may affect the number of victims in other crime categories in cases where a single (subsequently deceased) individual has been the victim of multiple types of crime.

As mentioned above, a proportion of outlaw biker and street gang violence is never recorded in official statistics–including in the PID registry. Nonetheless, while PID data may not capture all violent events, they do provide a reliable representation of the comparative levels of violence among the three groups.

Level of police proactivity affects the likelihood that a given crime will be officially recorded. It also varies over time. A direct measure of police attention would therefore be desirable. While no such measure is available, a reasonable proxy is found in the daily number of recorded cases of minor drug possession. Convictions for minor drug possession result in a warning, a fine or a maximum of two years imprisonment. These cases act as a suitable proxy for police attention because they indicate variations in the surveillance of target groups.

Table 2 provides descriptive statistics on HAMC individuals. Note that the table has six columns, two of which concern the Conflict Period and Extended Period, respectively. The remaining four columns concern four dates specifically chosen because they are the start and end dates of the two respective periods. The two columns concerning the Conflict Period and Extended Period include data on HAMC individuals who were PID-active at any point during the respective periods. Data for these columns are based on the subject’s status at his first PID registration. Data presented in the columns under the four specific dates indicate the subject’s status on those exact dates (age and number of prior convictions for acts of violence) or, in the case of annual-based data, on status during the years associated with the four dates (educational attainment, employment status, and family type). Gender is not included in the table since all individuals are male.

**Table 2:** Characteristics of HAMC individuals, by period and specific dates.

		Conflict Period	Extended Period	10 June 2007	6 July 2008	21 April 2012	30 June 2013
Age	Median	26.0	26.0	39.0	34.0	30.0	31.0
	Mean	28.7	28.5	37.7	34.1	33.7	34.0
	Variance	79.1	75.2	70.6	100.2	111.9	114.1
Number of prior convictions for acts of violence	Median	1	1	3	2	3	3
	Mean	3.5	3.3	5.6	4.5	4.5	4.6
	Variance	26.4	23.9	51.5	39.5	30.2	25.8
	Prevalence^A^	69%	68%	82%	78%	80%	81%
	Frequency^A^	5.1	4.9	6.8	5.8	5.7	5.7
Educational attainment	Primary school or lower	69%	70%	66%	63%	66%	69%
	Vocational/technical school	28%	27%	31%	33%	30%	27%
	Upper secondary level or higher	3%	3%	3%	4%	4%	5%
Employment status	Employed	33%	32%	65%	29%	34%	22%
	Unemployed	13%	13%	6%	5%	8%	8%
	Outside labor market	54%	55%	29%	65%	59%	70%
Family type	Married or cohabitating	14%	12%	23%	15%	15%	9%
	Single, living away from parents	66%	67%	66%	65%	64%	63%
	Single, living in parental home	20%	21%	11%	20%	20%	28%
Number of individuals		196	228	62	92	196	197

A: Prevalence is defined as the proportion of individuals in the data who are convicted of violence.

Frequency represents the mean number of convictions for violent crimes in the pool of active offenders (as distinct from the mean and median which are calculated for all HAMC individuals registered in PID).

The 198 and 278 cases of minor drug possession involved 82 and 107 individuals dealt with in 158 and 221 court cases across the two periods, respectively. As with Table 1, Table 2 includes information on the number of convicted individuals, court cases, and dates in order to give an impression of the context in which the cases of minor drug possession occurred and to illustrate their independence as observations. In the models below, the POLICE PROACTIVITY PROXY is coded into two levels (0; >0). Contrary to variation across the four dates, characteristics across the two periods vary only slightly. For example, while mean and median age at first PID registration are virtually identical for the two periods, they drop–sometimes dramatically–across the four specified dates.

## Method

The data used in this paper offer a rare opportunity to study whether violence committed by street gang members increases the likelihood that Hells Angels or their affiliates will commit violence. The sample is comprised of HAMC individuals where each individual in the sample has as many records as he has PID-active days during the period of study. Note that the sample grows over time as additional HAMC individuals are added to PID. Along with annual data on life circumstances, records on official criminal history are available to reduce the risk of selection bias.

Violence committed by individuals affiliated with the Hells Angels, Bandidos, and the four Copenhagen street gangs is included in the study and referred to as *HAMC violence*, *BMC violence*, and *street gang violence*, respectively.

As mentioned in the *Theory* section, the potentially reciprocal nature of gang violence has already been illustrated in previous studies of gangs. However, this is not the case with studies of outlaw bikers. Therefore, the current analysis focuses solely on how outlaw bikers react to outside violence and not on how the individual gang members react. This is reflected in the analytical setup in which the influence of the four Copenhagen street gangs only enters the regression models through a single variable which captures their violence, while individual records and multiple variables apply to the outlaw bikers. Furthermore, the analysis is focused on violence committed by individual outlaw bikers (the dependent variable) and not on an aggregate of their violence. This setup enables one to account for the influence of individual characteristics on the dependent variable. It should be noted that the latter does not imply that the influence of collective outlaw biker violence on the dependent variable is ignored–as explained below.

The effect of street gang violence on individual HAMC violence is estimated in a series of Cox proportional hazards regression models with multiple events using R’s survival package (Version 2.28–1). The Cox proportional hazards regression model was chosen because it is a conventional method for analyzing correlations in longitudinal data. Alternatively, the data could have been analyzed using a fixed-effects regression model. However, such models depend on the assumption that time-invariant unobserved variables are constant over time. Making such an assumption do not seem warranted in the current study.

While non-proportionality in Cox proportional hazards regression models might be thought to present a problem in some cases, note that in others “*A ‘significant’ nonproportionality may make no difference to the interpretation of a data set*, *particularly for large sample sizes*.” (inverted commas in original; page 142 in [78]). Non-proportionality may therefore make little or no difference if the variation in the estimates is small relative to their effect size or if it is based on a few outlaying observations [78].

The Cox regression models use one record per day for each individual–i.e. the unit of analysis is person-days. Furthermore, individuals are only included in the analysis as long as they are actively registered in PID. An individual is considered PID-active from his first date of registration until the day before he is dropped from the registry. In cases where subjects are registered in PID before the beginning of the Conflict Period and the Extended Periods, respectively, they are included in the analyses from the onset of both periods. However, only one PID data set (obtained June 2009) was available for examination of events occurring during the Conflict Period. Meanwhile, the analysis of events during the Extended Period was enriched by the inclusion of two additional PID datasets (obtained October 2012 and 2013). This has the implication that previously PID-active individuals, i.e., individuals appearing in the 2009 PID data, but not in the 2012 data, are only removed from the data analysis after October 2012 –regardless of the actual date in which they were dropped from PID. Consequently, no individuals are removed from the analysis during the Conflict Period.

The dataset is structured so that values on BMC violence, street gang violence and the Police Proactivity Proxy are shared by all HAMC individuals in the sample. To illustrate, if a hypothetical sample contained 100 HAMC individuals on Day D, then the value for each of these three variables would each be repeated in the dataset 100 times, i.e., one time for each individual on day D. These shared variables are *collective* in a double sense because they are shared by all individuals in the population and because they represent the sum of group activities, i.e., violence of BMC and street gang individuals and level of police proactivity.

A fourth collective variable, collective HAMC violence, deserves special attention. In the current analysis, it is used in some models as a control variable in the prediction of individual HAMC violence while not in others. Rationales for this are explained below. Furthermore, HAMC violence differs from the other three collective variables in another important way: In cases where an HAMC individual commits a violent act, this violent event is subtracted from collective HAMC violence on that particular day for that individual in order to avoid autocorrelation between it and the dependent variable. For all individuals other than the one committing the violent act, the control variable collective HAMC violence is identical on any given day.

The inclusion of multiple records for each individual deflates standard errors (thus inflating apparent statistical significance) in multivariate models via the introduction of autocorrelation. To deal with this problem, robust standard errors are estimated based on clusters of records from the same individual [79]. In this way, the robust errors take into account the fact that individual outlaw bikers are nested within a group.

The application of statistical controls on collective HAMC violence when predicting individual HAMC violence is justified by theoretical notions concerning competition for status within the group. However, controlling for collective HAMC violence has the implication of potentially controlling for a mediating causal variable on the causal path between the independent variable of interest (street gang violence) and the dependent variable (individual HAMC violence). This is because street gang violence may causally influence collective HAMC violence, which may then trigger or dampen additional street gang violence. And so the spiral continues. Controls that affect mediating variables in a causal path are problematic because they eliminate the causal relationship between the variables of interest [80]. In response to this challenge the models below are estimated with and without controls on collective HAMC violence.

## Results

Table 3 shows estimates of the effects of gang violence on violence committed by HAMC individuals derived from 24 separate Cox proportional hazards regression models with multiple events. As mentioned previously, the samples are derived from PID-active HAMC individuals, i.e., members of the Hells Angels and their affiliates connected to chapters in or around Greater Copenhagen. Data on BMC and street gang violence is based on activities in the same geographic region. Data on violent events is only included if an individual is found guilty in connection with the event. The independent variable of primary interest, *STREET GANG VIOLENCE*, is coded as an ordinal variable with three levels (0, 1, and >1). The third level, >1, indicates involvement in two or more violent acts by street gang members in a given period of time. There are two interrelated theoretical arguments for having applied this coding. The first theoretical argument is that if two gang members commit violence during the same short period of time, it seems more likely that their violence is related to gang activities than if it occurs across a broader spectrum of time. If activities are closely located in time, they are more likely to be related. Furthermore, gang related violence is more likely to be linked to the conflict with the Hells Angels than is individual violence. Here, it is important to bear in mind that not all violence committed by street gang members is related to the conflict with the Hells Angels. For instance, a gang member may commit violence in order to collect a drug debt or settle a personal dispute. The second theoretical argument is that a high level of violence generally seems more likely during a period of conflict than it would outside that period. This is why level three (>1) is of more theoretical interest than level two (1). The two theoretical arguments indicate that one would expect the association between level three of STREET GANG VIOLENCE and individual HAMC violence to be stronger than the association between level two STREET GANG VIOLENCE and individual HAMC violence. It turns out, that this is, in fact, the case.

**Table 3:** Estimates of street gang violence on individual HAMC violence in 24 Cox proportional hazards regression models with multiple events^A^.

Collective HAMC violence	Catchment period^B^	Full Model: All control variables^C^		Reduced Model: Only significant control variables^D^	
		Conflict Period	Extended Period	Conflict Period	Extended Period
Excluded	3	2.11**	1.71*	2.03*	1.71*
	5	1.18	0.95	1.15	1.05
	10	1.09	1.12	1.08	1.09
Included	3	1.96*	1.19	2.26**	1.21
	5	1.03	0.85	1.03	0.85
	10	1.04	0.98	1.07	1.02

* p<0.05

** p<0.01 (two-tailed z-test)

A: The number of observations varies with the period analyzed. Regression models on the Conflict Period include 196 HAMC individuals and 143 violent events across 206,626 person-day records. Regression models on the Extended Period include 228 HAMC individuals and 187 violent events across 321,904 person-day records.

B: The catchment period is the period during which the sum of events is calculated. In the 24 regression models, catchment periods of three, five and ten days are used.

C: The following control variables are included in the full regression models: STREET GANG VIOLENCE (two levels); BMC VIOLENCE, POLICE PROACTIVITY PROXY; PREVIOUS INDIVIDUAL VIOLENCE (two levels); AGE; EMPLOYMENT STATUS (two levels), FAMILY TYPE, EDUCATIONAL ATTAINMENT, SEASON, and WEEKDAY VS. WEEKEND.

D: The inclusion of control variables in the reduced regression models depends on whether they were significant in their respective full model regression counterpart. Therefore, different combinations of control variables are applied in the reduced regression models.

Since level three (>1) of STREET GANG VIOLENCE is of greater theoretical interest than level two (1) and because estimates for level two were all non-significant, only estimates for level three of STREET GANG VIOLENCE are shown in Table 3. *INDIVIDUAL HAMC VIOLENCE* is the dependent variable in all models and is coded dichotomously (0 and >0). In addition to STREET GANG VIOLENCE, the following variables are used to predict *INDIVIDUAL HAMC VIOLENCE*: *COLLECTIVE HAMC VIOLENCE*, *BMC VIOLENCE*, the *POLICE PROACTIVITY PROXY*, *PREVIOUS INDIVIDUAL VIOLENCE*, *AGE*, *EDUCATIONAL ATTAINMENT*, *FAMILY TYPE*, *EMPLOYMENT STATUS*, *SEASON*, and *WEEKDAY VS*. *WEEKEND*. Coding for all control variables can be seen in S1 Table with the exception of COLLECTIVE HAMC VIOLENCE which is described below.

Estimates are shown for events in the Conflict and Extended Periods, respectively, using different combinations of control variables. Models covering the Conflict Period include data on 196 HAMC individuals and 143 violent events across 206,626 person-day records. Models covering the Extended Period include data on 228 HAMC individuals and 187 violent events across 321,904 person-day records.

The first column in Table 3 indicates whether *COLLECTIVE HAMC VIOLENCE* is excluded or included in the model. The *POLICE PROACTIVITY PROXY* and the measures of collective violence committed by members or affiliates of the Hells Angels, Bandidos, or the four Copenhagen street gangs, respectively, are measured within three distinct *catchment periods* of three, five and ten days. These catchment periods indicate the number of days over which the sum of events is calculated. Where the catchment period is defined as three days and no violent events occur during the three-day period, the summed value for the relevant variable is 0. If one violent event occurs on each day of the three-day period, the summed value for the relevant variable is 3. COLLECTIVE HAMC VIOLENCE is coded into three levels (0, 1–2, and >2) while the POLICE PROACTIVITY PROXY and BMC VIOLENCE are both coded into two levels (0 and >0). The three-level coding of STREET GANG VIOLENCE was already described above. The coding is identical regardless of the length of the catchment period. These independent variables are *running sums* and the catchment period is the length over which they are calculated/running. The final two headers in Table 3 indicate whether the model includes all control variables (full model) or only those variables found to be significant in the full model (reduced model).

Very few if any HAMC individuals were registered as living with children, and those who do, do not seem to commit violence. Likewise, few HAMC individuals were registered as living with a partner (in a married, civil, or cohabitating partnership), or as having more than a high school degree. For these reasons, data on living with children was simply omitted from the models, while EDUCATIONAL ATTAINMENT was reduced to two categories (primary school or lower vs. more than primary school) and FAMILY TYPE was reconceived as Living with someone (a wife, romantic partner, parent) versus Living alone.

Only six of the 24 models depicted in Table 3 indicate a statistically significant (p<0.05) relationship between *STREET GANG VIOLENCE* and *INDIVIDUAL HAMC VIOLENCE*. Interestingly, these significant relationships are stronger both in terms of effect size and significance in models restricted to the (narrower) Conflict Period and only when variables are summed on the basis of a three-day catchment period. Movement from the full to reduced models (i.e., removal of non-significant control variables) has no substantive effect on the estimates obtained.

Separate models were estimated with different catchment periods because there are no theoretical grounds for assuming how long *STREET GANG VIOLENCE* is likely to influence others’ behavior. Yet Table 3 suggests that if *STREET GANG VIOLENCE* has an effect on *INDIVIDUAL HAMC VIOLENCE*, it is only identifiable using a three-day catchment period. Given this, the remaining discussion will focus on the models estimated with the three-day catchment period. Since violent responses by HAMC individuals to *STREET GANG VIOLENCE* are theoretically more likely to be concentrated in the Conflict Period than the Extended Period, the remaining discussion focuses its efforts here. Similarly, since the inclusion of all variables is more likely to reduce the risk of omitted variable bias, the remaining discussion focuses on the full models. Collectively, these criteria lead us to focus on two full models with three-day catchment periods and focus specifically on the Conflict Period. The first of these two models is significant at p<0.01 while the other is significant at p<0.05.

One of these two models contains the control variable *COLLECTIVE HAMC VIOLENCE* while the other does not. The two models are otherwise identical in terms of variables included. As mentioned, COLLECTIVE HAMC VIOLENCE is coded as an ordinal variable in three levels (0; 1; >1; with 0 as the excluded reference category). In the model with *COLLECTIVE HAMC VIOLENCE*, the estimated effect of *COLLECTIVE HAMC VIOLENCE* at the second level (1 violent event) seems unrealistically large (13.1; result not shown) and suspiciously significant (p<0.000). This unlikely result may reflect aforementioned potential problems of including mediating causal variables as model controls. Given this, models without this variable should be preferred. This restriction leaves us with a single Table 3 model on which to focus: the full model excluding *COLLECTIVE HAMC VIOLENCE* measured within the Conflict Period where STREET GANG VIOLENCE has a statistically significant effect on INDIVIDUAL HAMC VIOLENCE.

S1 Table describes the categorical independent variables in this model while S2 Table provides full model results. It should be recalled that individuals are represented multiple times in these data. Age is the only continuous control variable used in the model. The mean age is 33.2 years and the variance is 108.4.

In this model the POLICE PROACTIVITY PROXY approaches statistical significance (p = 0.059) at conventional levels and is positively associated with INDIVIDUAL HAMC VIOLENCE as would be expected.

Tests of proportional hazards based on Schoenfeld residuals indicate a failure to meet the proportional hazards assumption in the model. This is visible when reviewing plots based on the residuals for each independent variable. These plots are found in S1 Fig. However, the variation in the residuals over time appears neither systematic nor particularly large with the possible exception of that for *EMPLOYMENT STATUS* and *WEEKDAY VS*. *WEEKEND*. Furthermore, both variables are non-significant in the model results exhibited.

If the coding of *STREET GANG VIOLENCE* is reduced to two levels (0 and >0), its association with *INDIVIDUAL HAMC VIOLENCE* drops to non-significance in the model. Furthermore, recall that individuals appearing in the earlier, but not in the later PID data set are removed from the dataset the day before the later PID data is extracted from PID. If they are instead removed the day *after* their last appearance in the PID data set, the association between *STREET GANG VIOLENCE* and *INDIVIDUAL HAMC VIOLENCE* increases slightly in effect size and significance. This finding may reflect the less violent inclinations of the subjects removed who are probably older than those remaining in the population. This implies that the study’s results are robust and do not change substantially when outlaw bikers, who are deleted from PID, are removed at an earlier stage in the analyses.

The study’s methodology does not preclude the possibility that the causal direction of the estimated effect runs not only from gang members to HAMC individuals, but also in the opposite direction. However, this is somewhat less plausible given the fact that only violence within limited catchment periods is considered. The focus on violence within limited catchment periods also reduces the possibility that the results are a consequence of more general variations in the overall level of violence.

## Discussion and conclusions

Using a unique data set, this study examines and identifies a significant association at conventional statistical levels between violence committed by street gang members and violence committed by members of Hells Angels members and their support and puppet clubs in or around Greater Copenhagen. The association is identified within the context of an alleged conflict between the two groups. While the conflict may have been sparked by a personal dispute, subsequent violence seems to have arisen in connection with disagreements concerning control of the Copenhagen cannabis market and over subcultural status. The significant association between violence committed by gang members and that committed by HAMC individuals suggests that some of the violence committed by HAMC individuals is may be retaliation on behalf of the club. As such, the data support the cycle of gang violence model proposed by Decker [42] and expanded upon by Decker, Melde, and Pyrooz [17]. The study can therefore been seen as a test of whether their model is relevant in a specific European context involving groups that are not solely street gangs, i.e., outlaw motorcycle clubs. In other words, the results of the study indicate the presence of an inter-group micro-level process capable of generating reciprocal violence as predicted by Decker and colleagues in their cycle of gang violence model [17, 42]. The results suggest that violence committed by outlaw bikers has a dynamic character on a collective group level. The first violent act increases the risk of a second. Both of these tentatively identified characteristics of violence, i.e., its group nature and dynamic character, have been seen in North American street gang studies as well [46–47, 49]. The reciprocal violence might be both expressive and instrumental. As in North American street gang studies, the current study is unable to separate these forms of violence empirically [53].

More specifically, the elevated rate of violence committed by street gang members within previous three-day period was positively and significantly associated with violence committed by HAMC individuals during the Conflict Period (mid-2008 until early 2012) while controlling for a host of rival causal factors. As expected, the level of significance is higher when two or more violent gang events occur as compared to when only one occurs (p = 0.009 vs. p = 0.430). This indicates that an increase from a single recorded case of gang violence to more than one increases the risk of outlaw biker violence. As expected, the data indicate that the statistical association between violent acts was greater during the Conflict Period than it was in the Extended Period (mid-2007 until mid-2013).

This study has at least one noteworthy limitation. Two variables fail to meet the proportional hazards assumption. While both variables are non-significant in the model’s results, a parallel model (not shown) in which all controls are significant also results in a significant effect for *STREET GANG VIOLENCE* on *INDIVIDUAL HAMC VIOLENCE*.

An interesting observation is that the *POLICE PROACTIVITY PROXY*, which is based on recorded cases of minor possession of illegal substances, is positively associated with *INDIVIDUAL HAMC VIOLENCE and this* association approaches statistical significance (p = 0.059). This emphasizes the conventional wisdom that heightened police attention increases the rate of citation and arrest.

While the study indicates the presence of an inter-group micro-level process, intra-group micro-level processes leading to violence might also be present. In fact, given the high level of violence in the outlaw biker environment it would be surprising if they were not. Therefore, future research should test and compare the effects of multiple micro-level process within the same dataset in order to assess their relative importance. This is important because their relative effects may be concealed when tested separately, thereby obscuring theoretical development. Attention must, however, always be paid to whether the data are suitable for the tests required. For example, testing hypotheses on inter-group micro-level processes might be difficult with representative self-reported samples. This is because such samples are unlikely to catch sufficient amounts of data on interactions between groups. Likewise, demographic and criminal registry data, such as those used in the current study, may not be well suited for testing hypotheses on group affiliations and associated changes in personality and individual norms. The available data shapes the hypotheses we are able to test. Given this inherent weakness, it makes sense to study our research questions using diverse sources of data and conduct simultaneous tests on multiple hypotheses. It also makes sense to use network analyses and more qualitative methods to examine details of the individual criminal cases that are not easily observable using quantitative methods. Examples of the successful application of multiple methods are available in the literature [81]. Hopefully, the analysis of diverse sources of data with diverse methods will stimulate a nuanced theoretical development.

Societal reactions to the conflict may also provide a case for research in what has been termed a Janus-faced penal regime [82]. Scandinavian countries have relatively mild and benign penal regimes giving rise to the notion of “Nordic Exceptionalism” [83]. In the current context, an exit program for outlaw bikers administrated by the Danish police provides a good example of this Nordic Exceptionalism [84]. If applicants are found eligible and deemed properly motivated, the program will assist former bikers in finding places of residence, employment, education, and treatment for drug and alcohol addiction, as well as providing other forms of help. While such a program can be seen as not just benign, but even generous, some have noted that Scandinavian penal regimes also have a darker side or face that is more intrusive, punitive, and oppressive [82]. According to this perspective, Scandinavia sports a penal regime where an ethnocultural concept of citizenship “*…make certain categories of people such as criminal offenders*, *criminal aliens*, *drug offenders*, *and other perceived outsiders particularly vulnerable to deprivations and exclusion*.” (page 5 in [82]). In 2009, during the Conflict Period, the first of three sets of laws aimed at *gangs* in the broadest sense was adopted by the Danish parliament [85]. In this context, the term *gangs* included both members of street gangs and individuals associated with outlaw clubs like the Hells Angels and Bandidos. The first set of laws doubled the penalty for the possession of weapons and certain violent crimes if they were committed in connection with an event designated by police as a gang conflict. This first set of laws also made it possible to expel non-Danish citizens for the illegal possession of weapons if the violation was serious enough to result in a prison sentence. Note, however, that this was only relevant for a minority of both outlaw bikers and gang members, since the majority in both groups hold Danish citizenship. The second two sets of laws were adopted in 2014 and 2018, respectively [86–87]. Among a great deal of other things, the two sets of laws expanded the list of gang-related crimes that could result in a (doubled) penalty enhancement for those convicted in connection with gang conflicts,reduced the possibilities for parole for those same individuals as well as making possible to ban them from areas characterized by gang activity after completion of their sentences.

The conclusion of the current study calls for policy recommendations on the prevention of outlaw biker crime. Recently, the relevance of such recommendations has been emphasized. In a study of the policing of Norwegian outlaw bikers, two approaches are identified: one of confrontation and one of dialog [88]. Yet the two approaches mainly seem to reflect inherent styles of policing as opposed to competing policies based on evidence-based research. This might be because specific recommendations on the prevention of outlaw biker crime are very sparse. However, such recommendations do exist and include what has been termed a “whole-of-government approach” [3] or “holistic approach” [89]. Both approaches emphasize the importance of not only the criminal justice system, but also diverse governmental actors such as tax and customs officials and tattoo and liquor licensing authorities. Whether these approaches produce positive results is yet to be evaluated. From the current study, at least one additional recommendation can be derived and added to the limited literature. Outlaw motorcycle clubs exist in relation to other groups such as street gangs. This implies that the consequences of an intervention targeting an outlaw club cannot be isolated from the consequences this action has for other groups. A successful police intervention on one group might have positive or negative implications for another club and/or for law enforcement. For example, the incarceration of specific members of an outlaw biker chapter might, in theory, prevent retaliation against members of another group. Alternatively, it might tip the balance of power in favor of the other group. One policy recommendation for police interventions targeting outlaw motorcycle clubs is to take careful account of the possible consequences of their interventions on other criminal groups. Such an exercise may result in decisions to conduct simultaneous interventions on multiple groups.

On a more general level, the current paper and its identification of patterns of retaliatory outlaw biker violence are related to the ongoing deliberation on how to categorize outlaw motorcycle clubs in terms of their crime. First, the paper expands the gang literature on reciprocal violence to include outlaw motorcycle clubs. It warrants that some of the violence committed by outlaw bikers, regardless of whether it is expressive or instrumental, is of a similarly reciprocal nature to that carried out by gang members. In this way, the paper warrants that some outlaw bikers can be categorized as a type of gang. Second, while parts of the literature on outlaw bikers draw on (street) gang theory [3, 59], other parts of the literature draws on organized crime literature. It must be emphasized, however, that while some outlaw motorcycle clubs can be categorized to varying degrees as organized crime, others cannot [3, 62, 65]. Some definitions of organized crime point to the importance of violence as a means of goal attainment [90–91]. Whether the Copenhagen Hells Angels can be usefully classified as organized crime according to such definitions cannot be definitively settled in the current study. Yet, the study’s main finding that at least some of the violence committed by the Hells Angels and their affiliates is retaliation committed on behalf of their club, makes that classification more plausible. The current paper seems to warrant the categorization of outlaw biker clubs as both gangs and as organized crime. This has a few logical consequences. First, the term *outlaw motorcycle gang*—which has not been used previously in the current study but is widely used in the literature [3, 92–95], might actually be the most appropriate label. Second, in order to explain the complex crime patterns of outlaw bikers, it may be most beneficial to combine the literatures on organized crime and gangs. Likewise, it would probably be beneficial if researchers from both fields collaborate.

## Supporting information

S1 Table Bivariate relationship between categorical measures and Individual HAMC violence. S1 Table shows categorical measures and their bivariate relationships to Individual HAMC violence as used in the Cox proportional hazards regression model (196 unique individuals and 143 violent events across 206,626 person-day records) with all control variables except collective HAMC violence covering the Conflict Period (6 July 2008 to 21 April 2012).
(DOCX)

S2 Table Summary of Cox proportional hazards regression model predicting individual HAMC violence in the Conflict Period using a catchment period of three days. S2 Table provides a summary of the estimates derived from the Cox proportional hazards regression model. The regression model predicts individual HAMC violence. It is based on196 unique individuals and 143 violent events across 206,626 person-day records with all control variables except collective HAMC violence covering the Conflict Period (6 July 2008 to 21 April 2012).
(DOCX)

S1 Fig Schoenfeld residual plots. S1 Fig shows Schoenfeld residual plots for independent variables in the Cox proportional hazards regression model with all control variables except collective HAMC violence covering the Conflict Period (6 July 2008 to 21 April 2012).
(TIF)
