# Supplementary material for: Outlaw biker violence and retaliation
Source: PLoS One. 2019 May 8;14(5):e0216109. doi: 10.1371/journal.pone.0216109 (PMC6505941; doi:10.1371/journal.pone.0216109)
Supplement: S1 Table — shows categorical measures and their bivariate relationships to Individual HAMC violence as used in the Cox proportional hazards regression model (196 unique individuals and 143 violent events across 206,626 person-day records) with all control variables except collective HAMC violence covering the Conflict Period (6 July 2008 to 21 April 2012). (DOCX) [file pone.0216109.s001.docx]

**S1 Table.** Bivariate relationship between categorical measures and Individual HAMC violence

|  |  | Individual HAMC violence | |
| --- | --- | --- | --- |
|  |  | 0 | > 0 |
| Street gang violence | 0 | 168,901 | 124 |
|  | 1 | 29,781 | 10 |
|  | > 1 | 7801 | 9 |
| BMC violence | 0 | 166,566 | 125 |
|  | > 0 | 39,917 | 18 |
| Police Proactivity Proxy | 0 | 144,062 | 86 |
|  | > 0 | 62,421 | 57 |
| Previous individual violence | 0 - 1 | 83,430 | 33 |
|  | 2 - 5 | 64,535 | 49 |
|  | > 5 | 58,518 | 61 |
| Employment status | Employed | 51,388 | 18 |
|  | Unemployed | 16,881 | 13 |
|  | Outside the labor market | 138,214 | 112 |
| Family type | Living with someone | 136,410 | 102 |
|  | Living alone | 70,073 | 41 |
| Educational attainment | Primary school or lower | 139,980 | 103 |
|  | More than primary school | 66,503 | 40 |
| Weekday | Monday through Thursday | 117,926 | 85 |
|  | Friday through Sunday | 88,557 | 58 |
| Season | September through February | 108,380 | 72 |
|  | March through August | 98,103 | 71 |
