# Supplementary material for: Outlaw biker violence and retaliation
Source: PLoS One. 2019 May 8;14(5):e0216109. doi: 10.1371/journal.pone.0216109 (PMC6505941; doi:10.1371/journal.pone.0216109)
Supplement: S2 Table — provides a summary of the estimates derived from the Cox proportional hazards regression model. The regression model predicts individual HAMC violence. It is based on196 unique individuals and 143 violent events across 206,626 person-day records with all control variables except collective HAMC violence covering the Conflict Period (6 July 2008 to 21 April 2012). (DOCX) [file pone.0216109.s002.docx]

S2 Table. Summary of Cox proportional hazards regression model predicting individual HAMC violence in the Conflict Period using a catchment period of three days.

|  | Odds ratio estimate | Robust standard error^A^ | P-value |
| --- | --- | --- | --- |
| Gang violence: 0 vs. 1 | 1.21 | 0.24 | 0.430 |
| Gang violence: 0 vs. > 1 | 2.11 | 0.29 | 0.009 |
| BMC violence: 0 vs. > 0 | 0.65 | 0.17 | 0.012 |
| Police Proactivity Proxy: 0 vs. > 0 | 1.25 | 0.12 | 0.059 |
| Previous individual violence: 2 - 5 vs. 0 – 1 | 1.73 | 0.22 | 0.012 |
| Previous individual violence: > 5 vs. 0 – 1 | 1.11 | 0.21 | 0.620 |
| Age (continuous control variable) | 0.86 | 0.02 | 0.000 |
| Unemployed vs. employed | 1.16 | 0.63 | 0.813 |
| Outside the labor market vs. employed | 1.10 | 0.55 | 0.863 |
| Living with someone vs. single | 1.02 | 0.28 | 0.952 |
| Above primary school vs. primary school or lower | 1.41 | 0.18 | 0.056 |
| March through August vs. September through February | 0.98 | 0.16 | 0.940 |
| Monday through Thursday vs. Friday through Sunday | 1.23 | 0.20 | 0.305 |

Table note A: The robust standard error is for the odds ratio estimate and not for the logged odds ratio estimate.
