# Supplementary figures and images for: Outlaw biker violence and retaliation
Source: PLoS One. 2019 May 8;14(5):e0216109. doi: 10.1371/journal.pone.0216109 (PMC6505941; doi:10.1371/journal.pone.0216109)

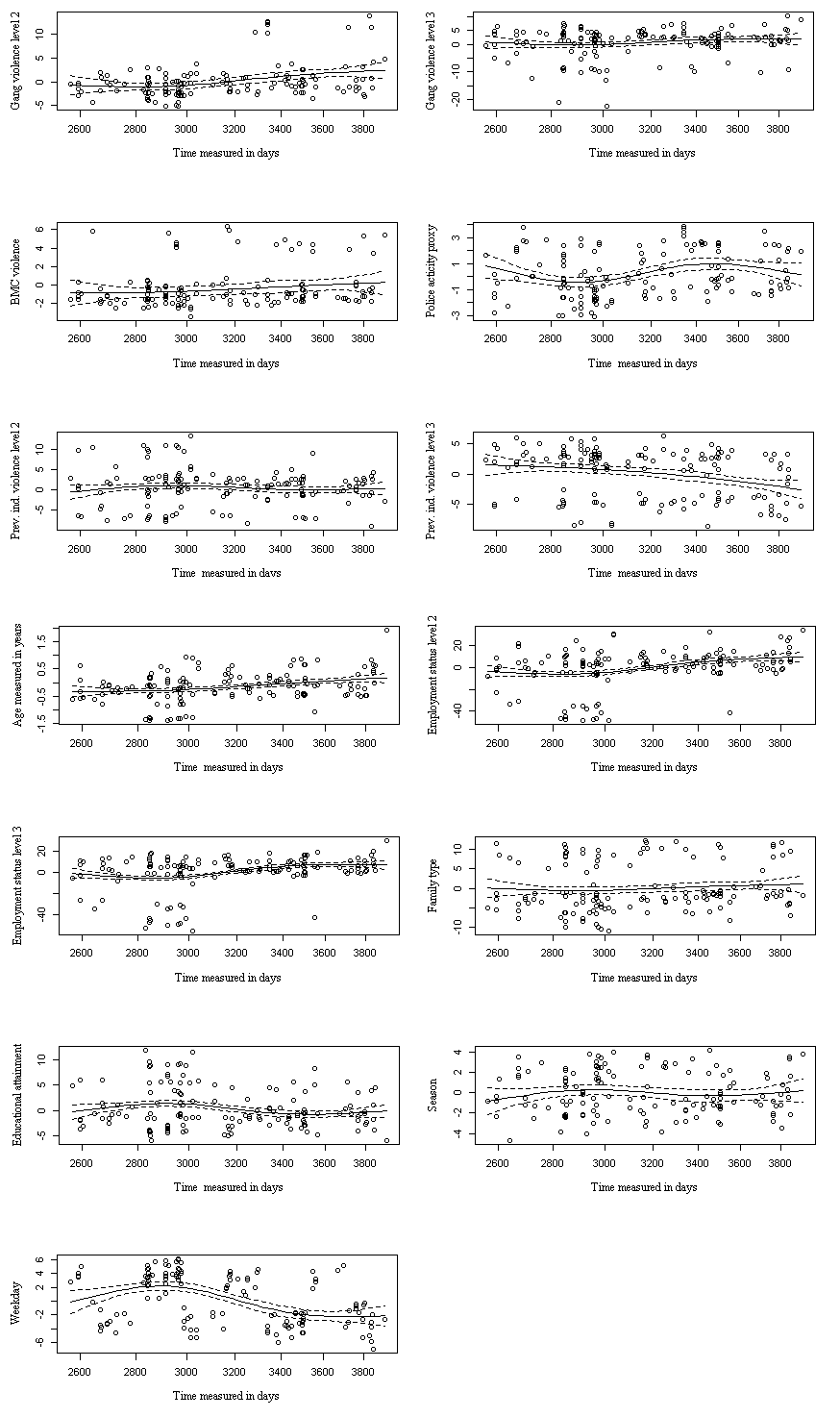

Supplement: S1 Fig — shows Schoenfeld residual plots for independent variables in the Cox proportional hazards regression model with all control variables except collective HAMC violence covering the Conflict Period (6 July 2008 to 21 April 2012). (TIF) [file pone.0216109.s003.tif]
